# Multiple causal pathways of high-quality development for semiconductor enterprises in China

**DOI:** 10.1371/journal.pone.0340629

**Published:** 2026-01-22

**Authors:** Shichuan Li, Xinjie Yang

**Affiliations:** Department of Global Business, Yeungnam University, Gyeongsan, Korea; Wuhan University of Technology, CHINA

## Abstract

As China’s economy undergoes transformation, enhancing the high-quality development of semiconductor enterprises has profound implications for overall economic growth. This study constructs a multi-factor configurational model influencing the high-quality development of semiconductor enterprises based on the Technology-Organization-Environment framework and conducts configurational analysis using the fuzzy-set qualitative comparative analysis method. The results indicate that high-quality development is a systematic process where no single factor is a necessary condition for the high-quality development of semiconductor enterprises. Five paths lead to high-quality development, which can be categorized into three types: innovation capability and enterprise size-dominated type, innovation capability and human capital level-driven type, and multi-factor linkage type. The study investigates the mechanisms of high-quality development from a structural perspective, providing insights into the synergistic effects of various factors and revealing the complex relationships underlying high-quality development.

## 1. Introduction

As a foundational and strategic sector, the semiconductor industry plays a critical role in supporting the development of other industries and forms the core of the electronic information sector [1]. At the micro level, semiconductor enterprises produce various microelectronic components, commonly known as “chips,” which are essential to all types of electronic products. These chips are central to information technology devices and are vital to national information security. Moreover, semiconductor manufacturing is among the most knowledge-intensive production processes today [2,3]. Chips now occupy a central position in modern manufacturing [4], playing a key role not only in emerging technologies such as cloud computing, blockchain, and artificial intelligence [5], but also in traditional sectors like automotive, home appliances, and industrial manufacturing [6].

Chip technology is fundamental to the development of Industry 4.0 and digital transformation [7]. As a pillar of numerous modern industries, the semiconductor sector has increasingly become a national strategic priority [8]. Data indicate that China’s semiconductor imports have surpassed oil, ranking as the country’s top import commodity [9]. Additionally, the upstream and downstream links in the semiconductor supply chain demonstrate high dependence on imported semiconductors [10].

However, China’s current participation in the global semiconductor value chain remains limited to low-value-added outsourcing services, with relatively weak involvement in core segments [11]. The global semiconductor value chain is unevenly distributed: design is concentrated in North America and Europe, while production and assembly are mostly located in Asia [5]. Meanwhile, international technological restrictions—particularly from the United States—have intensified challenges for China’s semiconductor industry [12]. These challenges manifest as a lack of core technologies, low innovation efficiency, and a constrained capacity to enhance supply chain capabilities. The industry faces production shortfalls, and the current self-sufficiency rate is low, failing to meet domestic demand [13].

As the world’s largest semiconductor market, China is undergoing a shift from high-speed to high-quality development [14], creating both opportunities and challenges for semiconductor firms. These enterprises play a central role in technological innovation, economic growth, and national defense [15]. High-quality development emphasizes improvements in the efficiency and quality of enterprise performance, thereby supporting long-term socioeconomic sustainability [16,17]. Enhancing productivity and identifying the key drivers of high-quality development are thus of critical importance to the ongoing transformation of the semiconductor sector.

A review of the literature shows that existing research on semiconductor firms has focused primarily on operational performance [18–20], technological innovation [21–24], supply chain management [25–28], and sustainable development [29–31]. However, most studies focus on the impact of single factors, with limited attention to the combined effects of multiple factors on high-quality development. As such, understanding how to enhance the synergistic capabilities of semiconductor firms has become a key issue in advancing sustainable development goals.

In the context of China’s economic transformation, high-quality development has become a guiding principle of national strategy and corporate governance. It marks a transition from high-speed to high-efficiency and high-value growth, focusing on innovation, coordination, sustainability, and inclusiveness. Rather than pursuing expansion alone, enterprises are encouraged to optimize quality and efficiency while maintaining social and environmental responsibility. This strategic shift is essential for China’s economy as it faces challenges such as resource constraints, rising production costs, and global technological competition. For technology-intensive sectors like semiconductors, high-quality development emphasizes innovation capability, digital transformation, and organizational resilience as key drivers of sustainable competitiveness. At the same time, high-quality development is not limited to economic performance—it is also closely tied to improving people’s quality of life. By promoting technological progress, environmental sustainability, and better employment opportunities, it supports the broader goal of “common prosperity.” In this study, we focus on the firm-level mechanisms that contribute to high-quality development, using the TOE framework and fsQCA to uncover how different configurations of technological, organizational, and environmental factors jointly enable this transformation.

The accelerated implementation of the “Digital China” strategy has provided strong policy and market support for technological advancement in the semiconductor industry. At the same time, external technological restrictions underscore the need to strengthen indigenous innovation and system resilience through internal industrial coordination—now a critical national strategic focus. In response to these challenges, this study adopts the Technology–Organization–Environment (TOE) framework to identify the antecedents of high-quality development in semiconductor firms. It applies fuzzy-set Qualitative Comparative Analysis (fsQCA) to explore the complex, configurational pathways driving such development. By addressing the current research gap, this study aims to offer a theoretical basis for future policy formulation and enterprise strategy, while contributing to the global positioning of China’s semiconductor industry through a deeper understanding of synergistic development mechanisms.

This study contributes to understanding how firms align with China’s national strategies such as the 14th Five-Year Plan and the “Digital China” initiative. By identifying five high-quality development pathways, particularly the “innovation–human capital-driven” configuration, this study demonstrates how digital capability enhancement supports the country’s 2035 goal of technological self-reliance.

## 2. Literature review and model construction

### 2.1. Literature review

In October 2017, China proposed the goal of achieving high-quality development, marking a strategic transition to a new stage of economic growth [32]. The theory of high-quality development originates from explorations into the determinants of economic growth [33]. It emphasizes not only economic expansion but also improved energy efficiency and the creation of a sustainable, livable environment [34]. As a development paradigm attuned to contemporary challenges, the high-quality development of enterprises diverges from traditional quality improvement, which primarily centers on products and services [35]. Instead, it integrates both economic and social dimensions [36], positioning enterprises as key actors in generating economic, social, and environmental value, thereby enhancing their sustainability [37].

Scholarly research on high-quality development has progressed notably in both theoretical and empirical domains. Theoretical studies have examined its content and implications through economic and bibliometric lenses, highlighting the necessity of balancing value and exchange value [38]. From China’s national context, the connotation of enterprise-level high-quality development has been elaborated in terms of target state, evaluation indicators, and enterprise growth [39,40].

In performance evaluation, scholars have investigated high-quality development across various levels: provincial [41,42], municipal [43–45], and within Free Trade Zones (FTZs) [46,47]. Research on influencing factors generally distinguishes between internal and external determinants. Internal factors—those controllable by enterprises—include the digital economy [14], innovation capability [48], green technology [49], and asset size [50]. External factors—beyond the control of individual firms—comprise environmental regulations [51], carbon emissions trading systems [52], the digital industry [53], and government subsidies. Most existing studies focus on the impact of single factors. However, in the semiconductor industry—a high-tech field with significant technical barriers—high-quality development requires multi-dimensional support [5]. The factors influencing enterprise development are not linear but systemic, involving complex interdependencies [54].

Building on prior scholarship, this study conducts a comprehensive multi-factor analysis of the high-quality development of semiconductor enterprises grounded in complex systems theory. Adopting the TOE framework, it categorizes influencing factors into technological, organizational, and environmental dimensions. Employing fuzzy-set qualitative comparative analysis (fsQCA), this research integrates qualitative and quantitative approaches to explore the multiple causal configurations that promote high-quality development. Ultimately, the findings aim to inform both enterprise strategies and policy decisions for advancing high-quality development.

Although the Technology–Organization–Environment (TOE) framework provides a comprehensive structure for analyzing the determinants of technological innovation and adoption, it mainly emphasizes the structural and contextual aspects of organizational behavior. To enrich its explanatory depth, this study integrates the Resource-Based View (RBV) and Co-evolution Theory as complementary perspectives.

This integration does not modify the variable composition of the TOE framework. Instead, it deepens the interpretation of its dimensions. Specifically, the RBV reinforces the organizational dimension by emphasizing internal resource capabilities such as human capital and R&D strength. Meanwhile, the Co-evolution Theory enhances the environmental dimension by introducing a dynamic adaptation perspective, highlighting the mutual adjustment between firms and their external environments.

Through this theoretical integration, the model maintains structural stability while gaining greater interpretive richness—offering a more holistic explanation of how firms achieve high-quality development through multiple configuration paths.

As shown in Table 1, this study retains the original TOE dimensions but enriches their meanings through theoretical integration. The organizational dimension now encompasses firm-specific capabilities highlighted by RBV, while the environmental dimension reflects dynamic adaptation as proposed by Co-evolution Theory. This integration strengthens the interpretive depth of the TOE-based analysis without altering its variable structure.

**Table 1:** Theoretical Integration of TOE, RBV, and Co-evolution Theory.

Theory	Core Focus	Key Constructs Reflected in TOE Dimensions	Main Contribution to This Study	Role in Theoretical Integration
Technology–Organization–Environment (TOE) Framework	Structural determinants of innovation adoption	Technology capability, organizational readiness, environmental pressure	Provides the baseline analytical framework for identifying key influencing factors	Foundation framework capturing contextual and structural influences
Resource-Based View (RBV)	Internal resources and capabilities as sources of competitive advantage	Reflected within the Organizational Dimension: R&D input, human capital, knowledge stock	Highlights how firm-specific resource endowments enhance innovation potential	Deepens the interpretation of TOE’s organizational dimension
Co-evolution Theory	Dynamic adaptation between firms and external environments	Reflected within the Environmental Dimension: policy pressure, market dynamics, external linkages	Emphasizes continuous mutual adjustment and adaptive learning	Deepens the interpretation of TOE’s environmental dimension

### 2.2. Model construction

Tornatzky and Fleischer first introduced the TOE framework in The Processes of Technological Innovation, identifying three dimensions—technological, organizational, and environmental—that shape technological innovation in enterprises [55]. The framework provides a balance between systematic analysis and flexibility. The technological dimension encompasses innovation capability, technology integration, and technological advancement. The organizational dimension emphasizes enterprise size, structure, and capabilities, while the environmental dimension includes factors such as government support and infrastructure [56]. By examining innovation from these three perspectives, the TOE framework effectively captures enterprise dynamics and has been widely applied in socioeconomic research [57]. Scholars argue that these conditions do not operate independently but interact with one another, allowing for configuration analysis based on the TOE model [58].

Semiconductor enterprises, situated in a high-tech and capital-intensive industry, are influenced by multifaceted factors in pursuing high-quality development. Hence, this study adopts the TOE framework to analyze the configuration of influencing factors across technological, organizational, and environmental dimensions. Prior research has constructed comprehensive causal models within the TOE framework to study the green supply chain decision-making of semiconductor firms, integrating multiple theoretical perspectives and validating its applicability [59]. Therefore, based on existing research and the characteristics of semiconductor enterprises, this study develops a framework for analyzing their high-quality development.

Technological Innovation Capability. According to Schumpeter’s innovation theory, innovation constitutes the essence of economic development [60] and serves as the primary driver of sustainable industrial growth [61]. In developing economies, technological innovation acts as a catalyst for long-term, sustainable advancement [62]. Through technological innovation, manufacturing enterprises can enhance production efficiency, reduce costs, and improve capacity [63]. Technology plays a decisive role in transforming total factor productivity, as enterprises enhance productivity by optimizing technology and management methods [64]. In the semiconductor industry, continuous innovation following Moore’s Law is essential for overcoming technological bottlenecks [65]. Patents, with their exclusivity and scarcity, represent key assets that confer competitive advantages and prevent technological imitation [66]. Empirical research confirms that patent intensity positively influences total factor productivity [67].

Digital transformation further strengthens technological capability. It plays a vital role in restructuring industries and driving global GDP growth [68]. Platformization and digital sharing improve resource utilization and foster deep integration of traditional industries with high-quality development goals [37]. Incremental innovation—minor improvements in existing products or processes—and radical innovation—fundamental technological breakthroughs—jointly contribute to competitiveness. Following prior studies, incremental innovation is measured by the ratio of utility model patents and process improvements, whereas radical innovation is reflected in invention patents, R&D intensity, and new product revenue [69]. Digitalization also enables the integration of artificial intelligence and the Internet to optimize production and deliver personalized services [70]. It not only drives innovation but also enhances industrial resilience through data platforms, promoting steady high-quality development [71]. Digitalization bridges information gaps, improves value acquisition efficiency [72], and enhances total factor productivity [73]. Moreover, it strengthens enterprises’ responsiveness to uncertainty [74] and boosts productivity via innovation and financial stability channels [75]. Government subsidies can further amplify these effects [76]. Overall, digital transformation enhances organizational resilience and infrastructure efficiency [77], underscoring the interdependence of multiple factors in the high-quality development of semiconductor enterprises.

Organizational Factors. The organizational dimension reflects internal resources, characteristics, and structural features that affect corporate integration [78]. Enterprise scale, structure, and resources exert a positive influence on development quality [79]. Human resource management significantly impacts organizational innovation capability; evidence from 35 UK manufacturing firms validates the effectiveness of HR systems in fostering innovation [80]. Organizational process innovation enables firms to acquire external knowledge, mitigating limitations in internal capabilities [81]. Additionally, organizational learning and internal governance mechanisms play key roles in advancing sustainable development [82,83].

Organizational Resilience. Within manufacturing enterprises, organizational resilience refers to the ability to recover, adapt, and transform under adversity [84]. Empirical research demonstrates that resilience positively affects firm performance [85] and continuity across industries such as pharmaceuticals [86]. High organizational resilience enables semiconductor firms to effectively manage external uncertainty, optimize resources, and minimize risk-related losses, thereby enhancing total factor productivity. Human Capital. Human capital is central to technological innovation and determines innovation outcomes [54]. Schultz emphasized its pivotal role in economic growth, as employee knowledge and technical skills directly contribute to enterprise performance [87]. A 10% increase in knowledge management correlates with a 9.3% rise in total factor productivity [88]. Human capital drives process optimization through spillover effects [89] and must interact with intellectual property systems to effectively enhance productivity [90]. Thus, high-level human capital supports innovation, improves production quality, and strengthens the structural competitiveness of semiconductor enterprises [91]. Enterprise Size. Firm size determines resource availability for innovation [92] and enhances the ability to identify and seize business opportunities [93]. Larger enterprises possess superior technological and supply chain advantages, enabling economies of scale and higher productivity [94]. In the semiconductor sector, firm size is significantly correlated with performance due to richer R&D resources [95]. Consequently, large semiconductor firms can reduce production costs, improve equipment utilization, and achieve market competitiveness, contributing to high-quality development.

Environmental Factors.Government support plays a crucial role in enterprise growth, particularly in strategically significant sectors such as semiconductors. Policy initiatives and industrial subsidies reduce technological dependence on foreign sources and promote domestic innovation [96]. Research indicates that government subsidies—both R&D and non-R&D—stimulate future productivity growth, especially in enterprises with high baseline efficiency [97]. Subsidies provide external funding to alleviate financial constraints and support long-term development [98]. However, excessive intervention may reduce innovation efficiency, forming a U-shaped relationship between subsidies and innovation [99]. Therefore, appropriate policy support can enhance R&D, optimize production processes, and reduce financial costs, collectively advancing the high-quality development of semiconductor enterprises.

### 2.3. Constructing the analysis framework diagram

**Fig 1:**
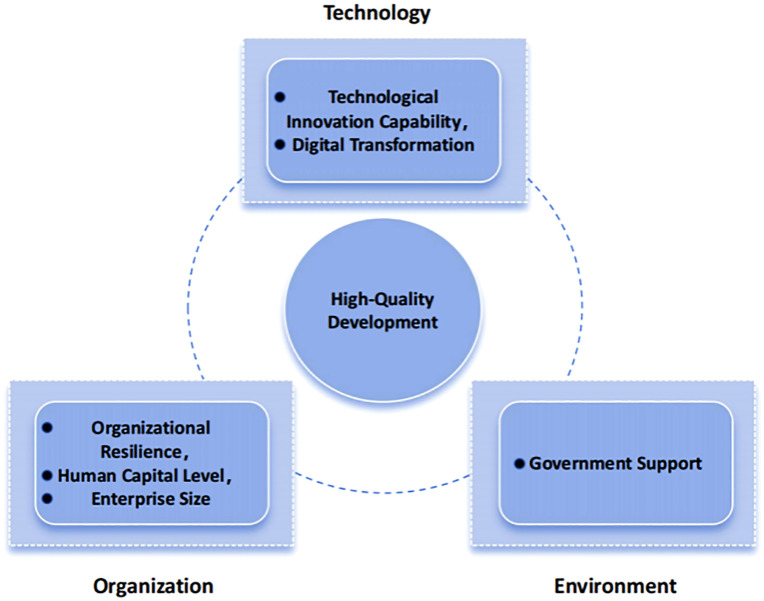
Theoretical Framework for the High-Quality Development of Semiconductor Enterprises.

Through analysis, it can be found that the six leading factors mentioned above will have an impact on the high-quality development of semiconductor enterprises. These factors influence each other, and the high-quality development of semiconductor enterprises may have a synergistic relationship at the technical level, organizational level, and environmental level. It is necessary to study the relationship between the six leading factors and the high-quality development of semiconductor enterprises, and explore the complex path of high-quality development of semiconductor enterprises from the perspective of configuration. Therefore, this study constructs a research model as shown in Fig 1.

## 3. Research methods and data sources

### 3.1. Qualitative comparative analysis

Qualitative Comparative Analysis (QCA) is a research method created by Charles Ragin, based on set theory and Boolean algebra. It integrates both qualitative and quantitative approaches to explore multiple causal relationships from a holistic and configurational perspective. This method posits that the influence of antecedent variables on outcome variables is not independent, but rather depends on the combinations of variables. The QCA method uses set theory to transform antecedent and outcome variables into a unified set for analysis, with particular focus on examining subsets [100]. Based on the distinction in processing variables, Qualitative Comparative Analysis (QCA) is divided into three types: crisp-set QCA (csQCA), multi-value QCA (mvQCA), and fuzzy-set QCA (fsQCA). Both csQCA and mvQCA are limited to handling categorical variables, while fsQCA allows for the assessment of variables with varying degrees of membership, ranging from full membership to full non-membership. This approach better captures subtle differences and is more suitable for handling complex, multi-variable scenarios [101]. fsQCA is suitable for analyzing small sample cases [102]. fsQCA is highly applicable to this study.

**Fig 2:**
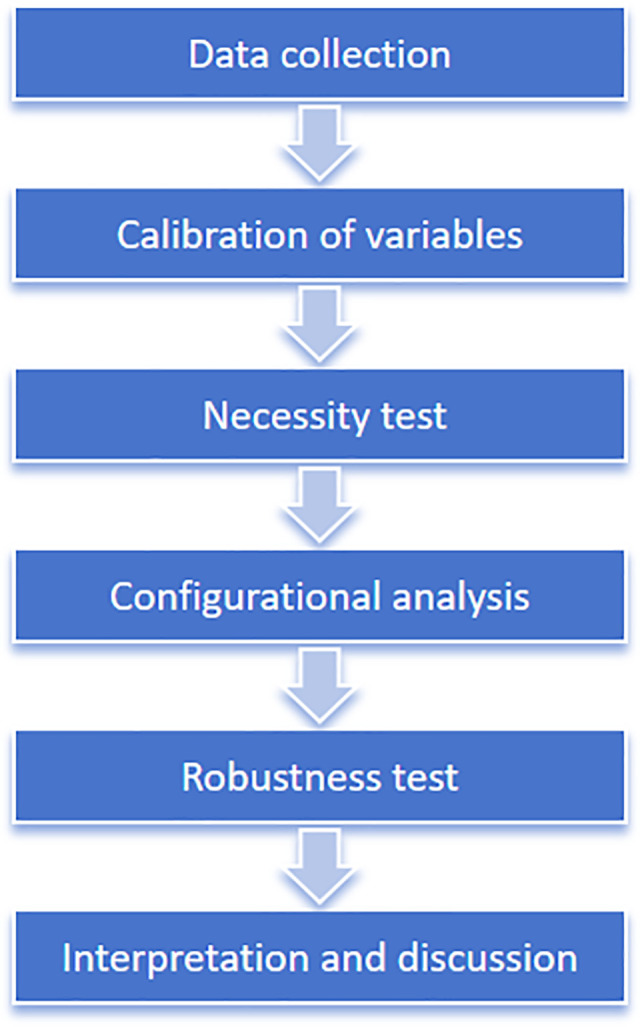
Research Process of fsQCA.

Therefore, this research adopts the fuzzy-set qualitative comparative analysis method to examine the impact of antecedent variables at the technological, organizational, and environmental levels on the high-quality development of semiconductor companies. Through comparative analysis across cases, this study aims to summarize the complex pathways leading to the high-quality development of semiconductor enterprises, providing both theoretical and practical guidance for strategic decision-making by governments and companies. The analytical procedure of this study follows a stepwise fsQCA approach. To enhance methodological transparency, Fig 2 illustrates the overall research process adopted in this study.

### 3.2. Data source and processing

#### 3.2.1. Data selection.

Semiconductor listed companies are typical representatives of the development of the semiconductor industry. Although they face a more complex environment, they are in an advantageous position in terms of R&D investment and business model, and are an important force in promoting the development of the semiconductor industry. This study selected semiconductor listed companies as the research object to explore the driving factors of the high-quality development path of semiconductor companies. This study uses the “2022 China Semiconductor Enterprise Research Report List” released by Jiwei Consulting as a reference. In the data collation, ST, PT and companies with missing data are eliminated, and finally 35 valid semiconductor companies are obtained as shown in Table 2. The data for this study comes from the annual reports of listed companies, CSMAR database, WIND database, CNRDS China Research Data Service Platform etc.

**Table 2:** Selected Enterprise Information.

Code	Company Name	Code	Company Name	Code	Company Name
600745	wingtech	688008	Montage Technology	688107	Shanghai Anlogic Infotech
603986	GigaDevice	300613	Fullhan	688766	Puya Semiconductor
600460	Silan	688798	Awinic	300474	JINGJIA MICRO
002049	Guoxin Micro	600360	Sino Microelectronics	688601	ETEK
688396	CR Microelectronics	603893	Rockchip	688508	Chipown
300373	Yangjie Electronic Technology	688536	3PEAK INCORPORATED	688699	Sunmoon Microelectronics
688728	Galaxycore	605111	NCE Power	603068	Beken Corporation
300223	Ingenic	300623	JieJie Microelectronics	688595	Chipsea Technologies
300672	Goke	688608	Bestechnic	300077	nationz technologies
688385	Fudan Micro	300327	Sino Wealth Electronic	300101	Chengdu Corpro Technology
300782	Maxscend Microelectronics	600171	Belling	688589	Leaguer
603160	Goodix	300458	Allwinner		

#### 3.2.2. Variable selection.

According to the previous analysis, this study uses total factor productivity as the outcome variable. The antecedent variables include technological innovation capability, digital transformation, organizational resilience, human capital level, enterprise size and government support. The calculation methods of each variable are as follows.

Outcome variable. High-quality development of enterprises is the micro-foundation of high-quality economic development. The core of high-quality development is to improve the total factor productivity of enterprises [103]. The total factor productivity of an enterprise is the comprehensive efficiency of converting resources into products [104]. A high value means that a high output can be obtained with the same input [105]. Total factor productivity has unique advantages and has important impacts on society, economy and individuals [106]. Most scholars use total factor productivity (TFP) as a proxy for indicators of high-quality enterprise development [49]. Total factor productivity (TFP) reflects a company’s balanced development capability. However, there is ongoing debate about which method provides the most accurate estimation of TFP. The DEA (Data Envelopment Analysis) method is suitable for macro-level TFP estimation. The OP (Olley-Pakes) method may encounter data instability issues, while the LP (Levinsohn-Petrin) method avoids using investment as a proxy variable, instead employing intermediate goods inputs, which are easier to obtain and can better address data missing issues. Therefore, this study refers to existing research by scholars and adopts the LP method [107]. The relevant data comes from the CSMAR database and the annual reports of listed companies.

Conditional variable. In the technology-driven dimension, the key indicators include technological innovation capability and digital transformation. Currently, there are two mainstream methods for representing a company’s technological innovation capability. These methods are the number of patents granted and the number of patent applications filed [108]. Compared to other participants in the industry, semiconductor companies tend to have a higher relative level of innovation when measured by patents [3]. Moreover, research has shown that the impact of invention patents on enterprise’s total factor productivity is greater than that of other types of patents [109]. Therefore, this article measures technological innovation capabilities by the number of invention patents independently obtained by semiconductor enterprises.

Digital transformation. Drawing on mainstream methods, this study employs text mining techniques to investigate the extent of digital transformation in enterprises [110]. We built a keyword thesaurus for digital transformation based on the five main aspects presented in the annual reports of companies, namely artificial intelligence technology (artificial intelligence, business intelligence, investment decision support system, etc.), big data technology (big data, data mining, data visualization, etc.), cloud computing technology (cloud computing, stream computing, graph computing, etc.), blockchain technology (digital currency, distributed computing, smart financial contracts, etc.), and the application of digital technology (mobile Internet, mobile payment, digital marketing, etc.). We used Python software to conduct quantitative analysis. The proportion of digital transformation keywords of listed companies to the total word frequency in the annual reports represents the degree of digital transformation of semiconductor companies. The value is expressed as the logarithm of the number of digital keywords plus 1. The data comes from the Wind database and the annual reports of listed companies.

The organizational driving dimension mainly includes three indicators: organizational resilience, human capital level and enterprise size. Organizational resilience refers to an organization’s ability to respond to shocks and recover after such shocks occur [111]. Drawing on scholars’ research [112]. This study conceptualizes organizational resilience as a two-dimensional structure characterized by efficient growth and low financial volatility. Long-term performance growth is measured by the cumulative increase in sales over a three-year period, while financial volatility is assessed using the standard deviation of monthly stock returns over one year. The overall organizational resilience is calculated based on these combined measures.

The level of human capital in a company determines its innovation capability. This study measures human capital intensity by the proportion of research and development (R&D) personnel relative to the total number of employees in the company [113]. Total assets are widely used as a measure of company size. The total assets of a company reflect the economic resources it holds and controls [114]. Therefore, this study uses total assets as a numerical measure of enterprise size and takes logarithmic processing of the data [115,116]. Environmental Driving Dimension. Subsidies provided by the government can help companies reduce their financial burden, speed up R&D progress and improve production capacity [117]. The government subsidy data in the annual report of the enterprise is used to represent policy support, reflecting the government’s support for semiconductor enterprises. The data is processed in logarithm [54].

#### 3.2.3. Variable calibration.

**Fig 3:**
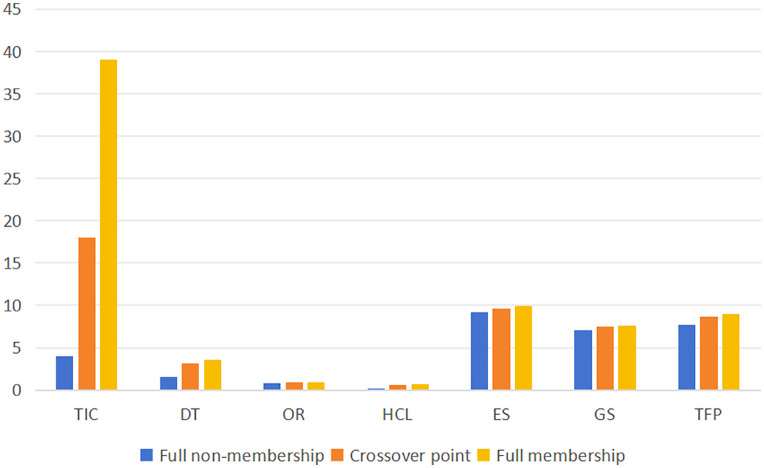
Calibration Anchor Histogram.

In the QCA research method, variable calibration is the process of assigning set membership to sample cases [118]. This study employs the direct calibration method to calibrate the variables of semiconductor enterprises, including technological innovation capability, digital transformation, organizational resilience, human capital level, enterprise size, government support, and total factor productivity. The calibrated set membership scores range from 0 to 1. Following the approach used by previous scholars, the fuzzy-set transformation of these variables is applied [119]. In this study, the full membership, crossover point, and full non-membership for each variable are set at 75%, 50%, and 25%, respectively. The variable descriptions and calibration anchor points are detailed in Table 3 and Fig 3.

**Table 3:** Calibration Anchors and Descriptive Statistics of Variables.

Variables	calibration			Descriptive Statistics			
	Full non- membership	Crossover point	Full membership	Minimum	Maximum	Mean	SD
TIC	4	18	39	1	250	34.860	47.058
DT	1.609	3.135	3.526	0.693	5.011	2.971	0.890
OR	0.843	0.87	0.882	0.838	0.907	0.871	0.019
HCL	0.183	0.622	0.737	0.149	0.893	0.573	0.222
ES	9.191	9.675	9.977	20.757	25.063	22.391	0.960
GS	7.077	7.457	7.649	16.050	19.780	17.329	0.819
TFP	7.724	8.694	8.974	7.500	10.490	8.633	0.654

Note: TIC = Technological Innovation Capability, DT = Digital Transformation, OR = Organizational Resilience, HCL = Human Capital Level, ES = Enterprise Size, GS = Government Support, TFP = Total Factor Productivity.

## 4. Empirical analysis

### 4.1. Necessity analysis

Before conducting configuration analysis, a necessity analysis is required. Necessity analysis is a separate step in the fsQCA method, which is used to determine whether the antecedent variable is a necessary condition for the occurrence of the result [120]. The analysis results are shown in Table 4, where the consistency of each individual causal condition is below 0.9. According to the testing standards proposed by scholars [121]. This indicates that technological innovation capability, digital transformation, organizational resilience, human capital level, firm size, and policy support do not constitute necessary conditions for the high-quality development of semiconductor enterprises, nor are they necessary conditions for their low-quality development.

**Table 4:** Analysis of necessary conditions.

Condition	PRESENCE		ABSENCE	
	Consistency	Coverage	Consistency	Coverage
TIC	0.700166	0.760192	0.319124	0.323142
~ TIC	0.376587	0.372271	0.763174	0.703603
DT	0.584760	0.569661	0.556542	0.505648
~ DT	0.492546	0.543571	0.526347	0.541743
OR	0.569299	0.580518	0.515098	0.489865
~ OR	0.499724	0.524942	0.558911	0.547564
HCL	0.585864	0.596738	0.517466	0.491564
~ HCL	0.500828	0.526713	0.575489	0.564460
ES	0.780232	0.785873	0.347543	0.326474
~ ES	0.331309	0.352526	0.772055	0.766157
GS	0.689674	0.691584	0.445234	0.416390
~ GS	0.418001	0.446871	0.670219	0.668241

Note: “~” indicates the negation of the condition.

### 4.2. Conditional configuration analysis

After conducting the necessity analysis, the truth table is used to test the sufficiency of causal condition configurations. Using the fsQCA 3.0 software, configurational analysis is performed on the data. From a set-theoretic perspective, configurational analysis examines the sufficiency of different combinations of causal conditions in producing various outcomes [121]. Referring to the research of scholars, we set the original consistency threshold, PRI threshold and case number threshold to 0.8, 0.75 and 1, respectively [122]. The analysis results will produce three solutions: complex solution, intermediate solution and simple solution. It is generally believed that the variables that appear in both the intermediate solution and the simple solution are core conditions, and the variables that only appear in the intermediate solution are marginal conditions. The results are shown in Table 5. There are three paths for the high-quality development of semiconductor enterprises, which can be summarized as innovation capability and enterprise scale-dominated, innovation capability and human capital level-driven, and multi-factor linkage. The path consistency of the individual configuration results is higher than 0.85, and the overall consistency is 0.941379, which exceeds the acceptable standard of 0.75. It can be seen that the five paths are sufficient conditions for the high-quality development of semiconductor enterprises, and the overall coverage is 0.602982, indicating that the five paths can explain about 60.3% of the high-quality development cases of semiconductor enterprises.

**Table 5:** Configuration for high-quality development in semiconductor enterprises.

Solutions						
Configuration	S1a	S1b	S2a	S2b	S3	
Technological Innovation Capability	●	●	●	●		
Digital Transformation		⊗		●	●	
Organizational Resilience	•		⊗	⊗	●	
Human Capital Level		⊗	●	●	●	
Enterprise Size	●	●	●		●	
Government Support	•	•	⊗	⨂	●	
Raw coverage	0.331309	0.213142	0.166759	0.21259	0.171728	
Unique coverage	0.094423	0.042518	0.006074	0.051905	0.053562	
Consistency	0.978793	0.974747	0.920732	0.869074	0.990446	
Overall solution coverage	0.602982					
Overall solution consistency	0.941379					

Note: ●indicates that the core condition exists, •indicates that the edge condition exists, ⨂indicates that the core condition is missing, ⊗ indicates that the edge condition is missing, blank indicates that the condition may or may not exist.

**Fig 4:**
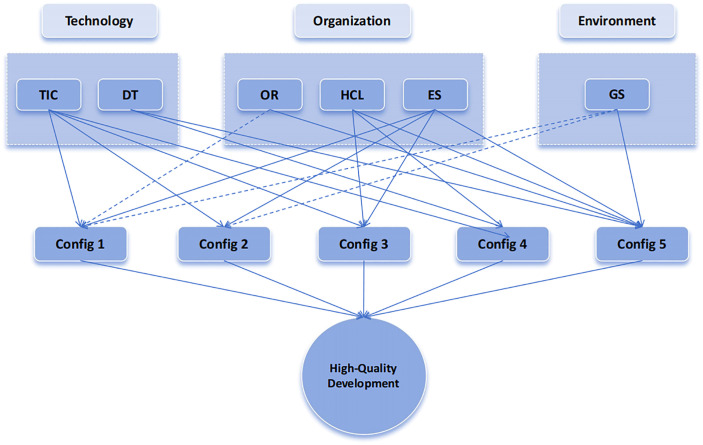
Configurational Analysis Framework.

This study presents the configurational results in the form of a configurational analysis framework, which facilitates a more intuitive display of core conditions (represented by solid lines) and peripheral conditions (represented by dashed lines), as illustrated in Fig 4.

1. Innovation capability and enterprise size-dominated type

Configuration S1a consists of Technological Innovation Capability* Enterprise Size* Organizational Resilience* Government Support. Specifically, Technological Innovation Capability and Enterprise Size are core conditions, Organizational Resilience and Government Support are marginal conditions, and Digital Transformation and Human Capital Level are optional. This path shows that semiconductor companies can achieve high-quality development with high-level technological innovation capabilities and leading enterprise asset scale, assisted by strong organizational resilience and appropriate government support. A typical enterprise following this path is China Resources Microelectronics Co., Ltd.

China Resources Microelectronics Co., Ltd has grown into a comprehensive semiconductor enterprise with scale effects through years of development and integration. The company boasts full industry chain capabilities, including chip design, wafer fabrication, and mask manufacturing. Its R&D achievements have been recognized as part of the central enterprise technology innovation products, earning it the National Science and Technology Progress Award (Second Class). Despite a revenue decline in the semiconductor industry due to cyclical adjustments, China Resources Microelectronics has demonstrated strong risk resilience, experiencing minimal impact. Furthermore, government support through subsidies and major projects has enhanced the company’s competitiveness in high-end power chips, thereby advancing its high-quality development.

Configuration S1b consists of Technological Innovation Capability * Enterprise Size * ~ Digital Transformation * ~ Human Capital Level * Government Support. Specifically, Technological Innovation Capability and Enterprise Size are core conditions, while Digital Transformation, Human Capital Level, and Government Support are marginal conditions. Organizational Resilience is optional. This pathway indicates that even though Digital Transformation and Human Capital Level may not be significantly motivating factors, semiconductor enterprises can still achieve high-quality development with high levels of technological innovation capability and substantial firm size, coupled with some degree of government support. A typical example of this pathway is Wingtech Technology Co., Ltd.

Wingtech Technology Co., Ltd. is a national high-tech enterprise. Its semiconductor division provides semiconductor, RFID and MiniLED manufacturing equipment, with more than 15,000 types and more than 800 new products added annually. However, in recent years, Wingtech Technology has frequently developed new businesses, and the company has paid more attention to production and cost control, and has not invested its superior resources in digital transformation and optimization of human capital. In 2023, with the help of the government, Wingtech Technology applied for and obtained the Customs AEO Advanced Certification. The Customs AEO system is advocated by the world customs. It provides customs clearance convenience for companies with excellent certification in many aspects. Among them, advanced certified companies are the highest credit rating awarded by the customs to companies, and are known as the “green pass” for international trade. The above factors also achieve high-quality development of the company.

1. Innovation capability and human capital level-driven type

Configuration S2a consists of Technological Innovation Capability* Human Capital Level* Enterprise Size* ~ Organizational Resilience* ~ Government Support. Specifically, Technological Innovation Capability, Human Capital Level and Enterprise Size are core conditions, Organizational Resilience and Government Support are marginal conditions, and Digital Transformation is optional. This path shows that the enterprise has a high level of technological capability and human capital level, and has a leading enterprise asset scale. Even if the enterprise is at a disadvantage in terms of organizational resilience and government support, it can still achieve high-quality development of semiconductor enterprises. A typical case of this path is Beijing Ingenic Integrated Circuit Co., Ltd.

Beijing Ingenic Integrated Circuit Co., Ltd. was established in 2005. It has continuously invested in R&D in processor technology and AI technology, and has a stable market share in the fields of intelligent monitoring, AIoT, biometrics, etc. In terms of talent training, the company cultivates a team of highly skilled talents, conducts advanced individual selection and commendation conferences every year, cultivates a “craftsman culture”, and at the same time develops recruitment channels and actively implements the talent-driven enterprise strategy to provide strong support for the high-quality development of the company.

Semiconductor Manufacturing International Corporation (SMIC) exemplifies the “innovation and human capital-driven” pathway. The company adheres to a strategy of technological self-reliance, focusing on the optimization and capacity expansion of mature process nodes (28nm and above), while continuously advancing in specialized process technologies. SMIC’s innovation is underpinned by a dual-track talent strategy: on one hand, it offers competitive salaries to recruit retired technical experts from leading Taiwanese semiconductor firms; on the other hand, it has established nine joint training platforms in collaboration with institutions such as Tsinghua University and the Chinese Academy of Sciences, and has launched the “Elite Program” in partnership with Fudan University. This program trains approximately 200 lithography process engineers annually, ensuring a steady pipeline of core technical talent. These initiatives have effectively supported SMIC’s technological accumulation and its differentiated competitive advantage. S2a shows a relatively low unique coverage, suggesting that it shares causal elements—particularly innovation capability and human capital—with other configurations. This overlap is common in fsQCA, where multiple sufficient pathways coexist and jointly account for the outcome [121,123].

Configuration S2b consists of Technological Innovation Capability* Digital Transformation* Human Capital Level* ~ Government Support* ~ Organizational Resilience. Specifically, Technological Innovation Capability, Digital Transformation, Human Capital Level and Government Support are core conditions, Organizational Resilience is a marginal condition, and Enterprise Size is optional. This path shows that the enterprise has a high level of technological innovation capability and human capital level, coupled with advanced digital transformation technology support, even if the enterprise has no government support and weak organizational resilience, it can still achieve high-quality development. A typical case of this enterprise is SiRuiPu Microelectronics Technology Co., Ltd.

SiRuiPu Microelectronics Technology Co., Ltd was established in 2012. It focuses on high-performance and high-quality integrated circuit products. Its products include signal chain analog chips, power analog chips and digital analog front-ends. The company provides competitive analog and embedded product solutions, which are widely used in communications, industry, medical health and other fields. The company believes that key technical personnel are the basis for the company to gain long-term competitive advantages. It provides competitive remuneration for technical researchers. At the same time, in order to improve the enthusiasm of employees, the company has successively implemented equity incentive plans and carried out multi-faceted cooperation with universities in order to jointly cultivate professional talents. In terms of digital transformation, SiRuiPu has demonstrated good digital capabilities in automated production, online customer platforms, digital resource management and Internet of Things integration.

1. Multi-factor linkage type

Configuration S3 consists of Digital Transformation* Organizational Resilience* Human Capital Level* Enterprise Size* Government Support, where Digital Transformation, Organizational Resilience, Human Capital Level, Enterprise Size and Government Support are the core conditions, and the Technological Innovation Capability condition is optional. This path shows that even if the technological innovation capability is not taken into account, when the enterprise has leading advantages in digital transformation, organizational resilience, human capital, enterprise scale and government support, the enterprise can make up for the shortcomings of technological innovation capability and achieve high-quality development of semiconductor enterprises. A typical case of this path is Montage Technology Co., Ltd.

Montage Technology Co., Ltd. is an international leader in data processing and interconnect chip design. The company has interconnect chips and Jintai server product lines, accurately judges market positioning and industry needs, and demonstrates high organizational resilience in the urgent situation of the global semiconductor supply chain, enabling Montage Technology to maintain growth in a highly competitive market environment. Since its establishment, the core team’s turnover rate has been 0, indicating that the company has excellent talent strategy and team building capabilities. At the same time, the company recruits overseas talents through a globalization strategy to provide strong support for the nationalization of its business. Montage Technology’s new controllable data center settled in Kunshan, Jiangsu, driving the development of the regional integrated circuit industry and gaining substantial support from the local government. In addition, the company has implemented an advanced supply chain management platform, which optimizes the supply chain process through digitalization and intelligence, and improves the transparency and efficiency of the process.

Yangtze Memory Technologies Co. (YMTC) serves as a representative case of the “multi-factor synergy” pathway. In 2023, the National Integrated Circuit Industry Investment Fund (commonly known as the “Big Fund”) injected an additional USD 1.9 billion, providing strong financial support for YMTC’s R&D and market expansion. Under favorable policy conditions, YMTC has fostered close collaboration with domestic manufacturers and suppliers, achieving partial import substitution for critical equipment such as lithography and etching machines, significantly enhancing its technological autonomy. Furthermore, its breakthroughs have spurred development across upstream and downstream segments—including packaging and testing, equipment, and materials—thereby contributing to the maturation of China’s memory chip ecosystem. This case highlights the vital role of multi-dimensional resource coupling in driving high-quality development.

In Pathway 1, large enterprises leverage their scale advantages and sustained resource investment to achieve technological progress primarily through incremental improvements in products and processes, exhibiting a strong pattern of path dependence. Pathway 2 is driven by high-quality human capital, characterized by strong absorptive capacity and cross-domain integration capabilities, which facilitates the ability to break away from established technological trajectories and enables breakthrough innovation. Pathway 3 reflects a multi-factor synergy, where the coordinated interaction of technological, market, and policy-related factors fosters both disruptive transformation and gradual accumulation, embodying a hybrid innovation pattern that combines “breakthrough” and “continuity.” To enhance the interpretability of the configurational results, representative firms were selected for each path to illustrate how different combinations of technological, organizational, and environmental conditions lead to high-quality development. Table 6 summarizes the core and supporting conditions for each case, as well as the key variables such as innovation capability, human capital, and digital transformation.

**Table 6:** Comparison of Representative Firms and Three Major Configurational Paths.

Path Type	Core Conditions	Optional Conditions	Representative Firms	Key Features of High-Quality Development
Path 1: Innovation Capability and Enterprise Size-Dominated Type	(TIC), (ES)	(OR), (GS)	China Resources Microelectronics Co., Ltd.; Wingtech Technology Co., Ltd.	Large firms leverage technological innovation and resource scale to achieve steady growth; organizational resilience and policy support enhance competitiveness despite industry fluctuations.
Path 2: Innovation Capability and Human Capital Level-Driven Type	(TIC), (HCL), (ES)	(DT)	Beijing Ingenic Integrated Circuit Co., Ltd.; SMIC; SiRuiPu Microelectronics Technology Co., Ltd.	High human capital and R&D investment enable technological self-reliance and breakthrough innovation, even with limited policy or organizational advantages.
Path 3: Multi-Factor Linkage Type	(DT), (OR), (HCL), (ES), (GS)	(TIC) (optional)	Montage Technology Co., Ltd.; Yangtze Memory Technologies Co. (YMTC)	Firms achieve high-quality development through coordinated interaction of digital transformation, talent, organizational adaptability, and government support, leading to systemic innovation and industrial upgrading.

### 4.3. Robustness test

This study follows the work of scholars and conducts a robustness test by adjusting the consistency threshold [124], increasing it from 0.8 to 0.85. The results show that the configuration obtained after adjustment is consistent with the previous one, as shown in Table 7. further validating the robustness of the study’s results.

**Table 7:** Robustness test.

Solutions						
Configuration	S1a	S1b	S2a	S2b	S3	
Technological Innovation Capability	●	●	●	●		
Digital Transformation		⊗		●	●	
Organizational Resilience	•		⊗	⊗	●	
Human Capital Level		⊗	●	●	●	
Enterprise Size	●	●	●		●	
Government Support	•	•	⊗	⨂	●	
Raw coverage	0.331309	0.213142	0.166759	0.21259	0.171728	
Unique coverage	0.094423	0.042518	0.006074	0.051905	0.053562	
Consistency	0.978793	0.974747	0.920732	0.869074	0.990446	
Overall solution coverage	0.602982					
Overall solution consistency	0.941379					

## 5. Discussions, implications and future research

### 5.1. Discussions

This study investigates the high-quality development of semiconductor enterprises through the lens of the TOE framework and the application of fuzzy-set qualitative comparative analysis (fsQCA). The results highlight the configurational nature of high-quality development, demonstrating that no single antecedent factor serves as a necessary condition. Instead, it is the interplay of various factors that drives success.

The findings reveal five distinct pathways to high-quality development, categorized into three primary types: innovation capability and enterprise size-dominated type, innovation capability and human capital level-driven type, and multi-factor linkage type. These pathways underscore the diversity of developmental strategies semiconductor enterprises can adopt. The dominance of the S1a pathway highlights the critical role of technological innovation capability, emphasizing the need for firms to focus on R&D investment, advanced equipment, and technology upgrades. The observed substitution effect between S1a and S1b pathways further underscores the flexibility enterprises have in choosing their developmental trajectories based on specific contexts.

Another significant finding is the non-linear relationship between resource allocation and development outcomes. Excessive allocation of resources can lead to redundancy, reducing efficiency and performance. This insight challenges traditional perspectives on resource-based growth and underscores the importance of optimal resource configuration. Enterprises must judiciously allocate resources to avoid inefficiencies while ensuring critical factors like innovation and human capital are adequately supported. The findings provide practical implications for realizing China’s vision of high-quality and innovation-driven growth by enhancing digital capabilities and human capital synergy.

### 5.2. Theoretical contributions

This study makes several theoretical contributions. First, This study advances the TOE framework by embedding resource-based and co-evolutionary insights, highlighting how internal capabilities (resources, innovation, and human capital) interact dynamically with external environmental forces. These additions expand the framework’s applicability to technology-intensive industries, such as semiconductors. Second, the study extends the resource-based view (RBV) and dynamic capabilities literature by identifying innovation capability, organizational resilience, and talent management as pivotal factors for sustaining high-quality development. By theoretically extending the TOE framework through the integration of RBV and Co-evolution Theory, this study maintains structural simplicity while enhancing interpretive richness, offering a multi-perspective understanding of high-quality development in dynamic contexts. Third, the configurational approach adopted here shifts the focus from linear cause-effect relationships to complex interdependencies among factors, advancing theoretical discourse on competitive advantage and innovation in high-tech sectors.

The findings also highlight the context-specific nature of high-quality development, emphasizing the importance of tailoring strategies to align with organizational and environmental conditions. This contributes to a more nuanced understanding of the dynamic interplay between resources and outcomes in technology-driven industries.

This study extends the applicability of the Technology–Organization–Environment (TOE) framework to the semiconductor industry by incorporating organizational resilience, human capital, and digital transformation, complementing the resource-based view (RBV). The findings reveal multiple pathways to achieving high-quality development, thereby challenging traditional linear causal logic and resonating with the systemic interactions and path dependence emphasized in industrial co-evolution theory. The identified typical configurations align with national initiatives such as “Digital China” and the 2035 goal of technological self-reliance, highlighting how firms achieve strategic synergy through enhanced digital capabilities and resilience.

Among the five high-quality development pathways identified in this study, several strongly align with China’s 14th Five-Year Plan and the national strategy of building a “Digital China.” In particular, the “innovation–human capital-driven” pathway emphasizes the critical role of R&D talent and technological competence in coping with environmental uncertainty. During the 14th Five-Year period, high-quality development was established as a core theme, with scientific and technological innovation recognized as the key driver of quality transformation. Talent has been positioned as the primary resource for enterprise growth and national competitiveness. The country is currently accelerating efforts to build a globally influential talent hub, promoting the tiered cultivation of high-level talent, and emphasizing the strategic value of core technical groups such as outstanding engineers. This study advances prior work by integrating the Resource-Based View (RBV) and co-evolutionary theory into the TOE framework, thereby offering a multi-theoretical explanation of high-quality development and revealing multiple sufficient pathways through fsQCA — a perspective rarely applied in the Chinese semiconductor context.

### 5.3. Practical implications

This study provides actionable implications for strategic management within the semiconductor industry. Firms are advised to assess their positioning along distinct developmental trajectories and adopt context-specific strategies. Enhancing technological innovation capability remains essential, particularly through targeted R&D in advanced process nodes, AI-assisted chip design, and energy-efficient architectures. Concurrently, investment in digital infrastructure—such as manufacturing execution systems (MES), real-time data analytics, and automated logistics—is critical for improving operational agility.

Human capital development is another pivotal factor. Firms should strengthen university–industry linkages, implement continuous technical training, and adopt structured incentive mechanisms to attract and retain skilled personnel. For firms following the S2b pathway, prioritized digital transformation initiatives—such as ERP/CRM upgrades and deployment of intelligent customer-facing platforms—can significantly enhance responsiveness and competitiveness.

From a policy perspective, the findings underscore the need for targeted instruments including R&D tax incentives, innovation grants for SMEs, and public–private co-investment in foundational semiconductor infrastructure. In addition, robust intellectual property protection and institutional support for collaborative innovation ecosystems are essential to foster sustained industry-wide advancement.

### 5.4. Future research

Although fsQCA effectively identifies multiple pathways to high-quality development, it cannot fully capture the dynamic interactions between firm strategies and external environments—particularly critical in the fast-evolving semiconductor industry. Future studies may consider dynamic approaches, such as panel QCA or mixed methods combining QCA with event history analysis, to explore how configurations evolve over time and to better understand causal complexity and path dependence. Longitudinal case studies can also help validate the stability and transformation of identified pathways. Finally, as the current findings reflect statistical associations, causal interpretations should be made with caution. Future research could leverage natural experiments, exogenous shocks, or stronger identification strategies to enhance causal inference. It should be noted that the relationships revealed by the fsQCA method represent configurational associations rather than direct causal effects. The identified paths indicate sufficient conditions for achieving high-quality development, rather than necessary or deterministic relationships. Future studies may employ longitudinal or experimental designs to verify causal mechanisms.

## 6. Conclusions

The Chinese government proposed a new concept of “promoting changes in the quality, efficiency and driving force of economic development and improving total factor productivity”, and improving total factor productivity is an important path for China to achieve high-quality and sustainable economic development. Based on the TOE framework, this study constructs a theoretical framework for the high-quality development of semiconductor companies from an overall perspective of technology, organization, and environment. It selects technological innovation capabilities, digital transformation, organizational resilience, human capital level, enterprise size, and government support as antecedent variables, and uses the fsQCA method to analyze the configuration path of the high-quality development of semiconductor companies. This study enriches the theoretical research of the TOE framework in the field of high-quality development, and at the same time innovatively reveals the configuration path of the micro-level high-quality development of semiconductor companies.

This study draws the following conclusions: No single antecedent factor is a necessary condition for the high-quality development of semiconductor enterprises. Achieving high-quality development requires configurational pathways that operate on multiple levels, reflecting the complexity of these developmental routes. While some semiconductor enterprises may lack advantages in certain areas, they can still attain high-quality development through configurational effects. Technological innovation capability plays a central role in the high-quality development of semiconductor enterprises, while firm size and human capital level are also crucial. Enterprises should strategically enhance these variables to support their high-quality growth.

The five pathways to high-quality development can be categorized into three types: innovation capability and enterprise size-dominated type, innovation capability and human capital level-driven type, and multi-factor linkage type. These pathways have an overall coverage of approximately 60.3%, capturing the majority of high-quality development cases among semiconductor enterprises, demonstrating strong explanatory power. The S1a pathway is predominant, with S1a and S1b representing an overall substitution effect, allowing enterprises to choose a development path that suits their specific context. The configurational results indicate that greater resource input does not always yield better outcomes. Excessive resource allocation can lead to resource redundancy, underscoring the need for semiconductor enterprises to allocate resources judiciously.
